# Whole genome sequencing and *de novo* genome assembly of the Kazakh native horse Zhabe

**DOI:** 10.3389/fgene.2024.1466382

**Published:** 2024-10-21

**Authors:** Tolegen Assanbayev, Rakhmetolla Akilzhanov, Tlekbol Sharapatov, Rakhimbek Bektayev, Diana Samatkyzy, Daniyar Karabayev, Aidana Gabdulkayum, Asset Daniyarov, Saule Rakhimova, Ulan Kozhamkulov, Dos Sarbassov, Ainur Akilzhanova, Ulykbek Kairov

**Affiliations:** ^1^ Department of Zootechnology and Veterinary Medicine, Toraighyrov University, Pavlodar, Kazakhstan; ^2^ Laboratory of Bioinformatics and Systems Biology, Center for Life Sciences, National Laboratory Astana, Nazarbayev University, Astana, Kazakhstan; ^3^ Laboratory of Genomic and Personalized Medicine, Center for Life Sciences, National Laboratory Astana, Nazarbayev University, Astana, Kazakhstan; ^4^ Faculty of Natural Sciences, L.N.Gumilyov Eurasian National University, Astana, Kazakhstan; ^5^ School of Sciences and Humanities, Nazarbayev University, Astana, Kazakhstan

**Keywords:** Kazakh horse, oxford nanopore technologies (ONT), *de novo* assembly, Kazakhstan, whole genome sequencing (WGS)

## Introduction

The horse (*Equus caballus*) is a domesticated animal with great significance in human civilization and history, having played a crucial role in transportation, agriculture, and warfare. Over millennia, intentional breeding has resulted in the creation of approximately 500 distinct horse breeds, each selected for specific performance qualities, appearance, and behavior (Petersen et al., 2013). The earliest evidence of horse domestication dates back to the Eneolithic Botai culture (3500 BCE) in prehistoric Northern Kazakhstan, where horses continue to hold cultural significance (Outram et al., 2009; Levine, 1999; Sarbassova, 2015). Although domestication in Botai occurred independently of the main domestication path, horses have been an essential aspect of steppe pastoralism in the region of modern Kazakhstan since the Bronze Age (Kyselý and Peške, 2022; Frachetti and Benecke, 2009; Outram et al., 2012). As a result, traditional selection over hundreds and thousands of years has shaped the Kazakh horse breed (Kabylbekova et al., 2024).

**FIGURE 1:**
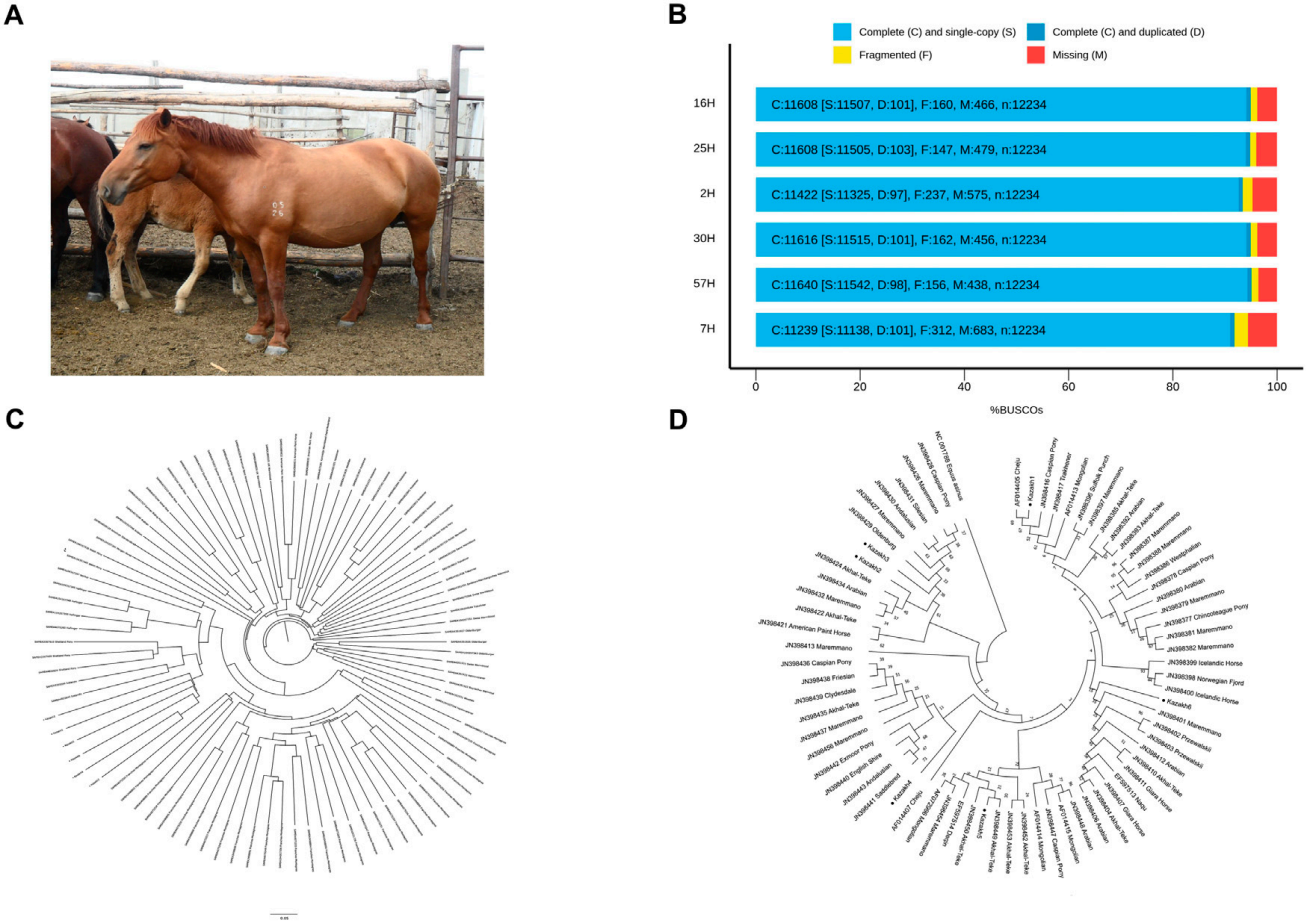
*De novo* genome assembly of Kazakh horse of Zhabe type. **(A)** Representative image of Kazakh horse of Zhabe traditional type. **(B)** BUSCO assessment results: each column represents the percentage of identified BUSCO genes in genome samples. **(C)** Genomic relationships between the Kazakh horse and other horse breeds shown by a neighbor-joining phylogeny tree (samples 2–57H are respectively presented as Kazakh1-6). **(D)** Neighbor-joining bootstrap consensus tree reconstructed from the mtDNA control region sequences of Kazakh horse and other horse breeds (samples 2–57H are respectively presented as Kazakh1-6).

Zhabe is an intrabreed type of Kazakh horse that originated in Western Kazakhstan and is currently used throughout the country (Figure 1A). This type is known for its strong, slightly rough constitution and high endurance. Horses of this type are characterized by a coarse head, a short fleshy neck, a wide and deep body, a broad back, a muscular croup, and strong, bony legs. They also have a thick, long mane and tail, short fetlocks on the legs, and dense skin. Their colors are typically bay or dark red, but can also be mousey, gray, or black (Dmitriev and Ėrnst, 1989). In state farm conditions, Kazakh horses, including Zhabe, have been selectively bred for increased size and weight. They are well-adapted to traditional Kazakh methods of seasonal pasturing and are bred in herds, even inharsh winter climatic conditions, to produce working horses, meat, and milk (Omarov et al., 2019).

Previous studies have characterized Kazakh horses using array-based genotyping, RNA-seq, and WGBS-seq (Pozharskiy et al., 2023; Liu et al., 2018; Yu et al., 2021; Liu et al., 2023). This study presents the first high-quality genome assemblies for six Kazakh horses of the Zhabe type, providing a valuable resource for genetic research and comparative genomics. Conserving genetic diversity is vital for the present and future maintenance of the valuable traits of the breed (Bruford et al., 2015). It is also widely acknowledged that comprehensive molecular genetic data characterizing inter- and intraspecies diversity is important for the efficient management of genetic resources economically important animal varieties (Ruane, 2000; Simianer, 2005; Toro et al., 2009). Here, we present six new *de novo* genome assemblies, generated using Oxford Nanopore Technology, for Kazakh horses of the Zhabe traditional type.

## Materials and methods

### Sample collection

Peripheral blood samples from six horses (2H, 7H, 16H, 25H, 30H, and 57H) were collected in 1 mL volumes at “Akzhar Ondiris” horse farm (51°32′07.4″N 77°27′16.9″E) in Pavlodar region of Kazakhstan (Figure 1A). All samples were anticoagulated with EDTA and refrigerated at 4°C. The phenotypic characteristics of these horses are detailed in Supplementary Table S1. Genomic DNA was extracted from the samples using Illustra Blood Kit (Cytiva, United State) and Gentra Puregene Blood Kit (Qiagen, Germany) following the manufacturers’ protocols. The concentration and quality of the extracted DNA were checked using a Qubit fluorometer (Invitrogen, United State), a Nanodrop 2000 spectrophotometer (Thermo Scientific, United State), and 1% agarose gel electrophoresis. This high-molecular-weight DNA was then used for library construction and subsequent Nanopore sequencing.

### Library construction and genome sequencing

To generate Oxford Nanopore long reads, 3 µg of genomic DNA was randomly sheared to obtain a target size of 20 kbp using g-TUBE (Covaris, United State) and processed according to the Ligation Sequencing Kit (SQK-LSK110) protocol (Oxford Nanopore Technologies, United Kingdom). For genome sequencing, at least 1 µg of sheared DNA from each sample was utilized for library construction. DNA fragments were repaired using NEBNext FFPE Repair Mix (New England Biolabs, United State). End repair and A-tailing were performed using the NEBNext End Repair/dA-Tailing Module kit (New England Biolabs, United State), followed by ligation of Oxford Nanopore sequencing adapters with the NEBNext Quick Ligation Module (E6056) (New England Biolabs, United State). The constructed libraries were sequenced on R9.4.1 flow cells of PromethION sequencer (Oxford Nanopore Technologies, United Kingdom) for 72 h. Basecalling of the raw signal data was performed using Guppy v.5.1.13, which also trimmed adapters and removed low-quality sequencing reads with a Q-score below 9.0. All DNA samples were sequenced with an average coverage of 26X. A summary of the sequenced reads is provided in Supplementary Table S2.

### Genome assembly and evaluation

Draft assemblies were produced using one round of Flye v.2.9.2 (Kolmogorov et al., 2019), followed by a polishing round with Oxford Nanopore Technologies (ONT) reads using Medaka v.1.11.1 (https://github.com/nanoporetech/medaka). To evaluate the quality of the final assemblies, we aligned the ONT contigs to EquCab3.0 reference genome assembly (NCBI Accession No. GCF_002863925.1) and assessed them with QUAST v.5.2.0 (Gurevich et al., 2013). Considering the advanced sequencing ability of ONT, the longest contig among the assembled genomes was 92.32 Mb, and the largest contig N50 was 28.26 Mb. The completeness of the genome assemblies was further assessed using BUSCO v.5.4.6 (Simão et al., 2015), which compared the genome against the *laurasiatheria_odb10* database containing 12,234 orthologous genes. BUSCO assessment scores ranged from 93% to 95% (Figure 1B; Table 1), indicating high completeness for the obtained assemblies.

**TABLE 1:** QUAST metrics and BUSCO assessment results of the sequencing data.

	2H	7H	16H	25H	30H	57H
Number of contigs	4210	3459	4405	4662	4247	4180
Contig N50 (bp)	25,995,087	26,306,096	25,978,232	25,961,887	19,984,809	26,032,890
Largest contig (bp)	75,750,993	67,042,342	89,046,621	62,359,761	92,322,303	63,939,966
Total length (bp)	2,551,255,215	2,534,155,455	2,590,139,805	2,602,523,862	2,608,619,890	2,600,392,272
GC content (%)	42.33	42.25	42.55	42.65	42.63	42.65
Genome fraction (%)	96.037	95.937	96.115	96.106	96.116	96.032
Complete BUSCOs (%)	93.4	91.8	94.9	94.8	94.9	95.1

## Data analysis

### Variation statistics

To identify SNVs and indels, the wf-human-variation Epi2Me Labs pipeline from ONT (https://github.com/epi2me-labs/wf-human-variation) was used. Samples were analyzed using Clair3 v.1.0.4, which identified small variants in ONT reads (Supplementary Table S3). The number of identified SNVs ranged from 6,336,129 to 7,101,556, while the number of identified indels ranged between 549,718 and 820,662 across samples.

### Comparative genomics

Phylogenetic analysis and tree construction were performed using VCF-kit v.0.2.6 (Cook and Andersen, 2017) and MEGA software v.11.0.13 (Tamura et al., 2021). The neighbor-joining tree was constructed using 1,331,674 mutation points from a merged VCF file (Figure 1C) containing data from Kazakh horses and 88 additional horse samples (Jagannathan et al., 2019) deposited in the European Nucleotide Archive (ENA) database (https://www.ebi.ac.uk/ena/). At the autosomal genetic level, Kazakh Zhabe horses formed a distinct cluster and a separate group compared to other horse breeds. Additionally, a multiple sequence alignment of all mitochondrial D-loop sequences was performed in MEGA using the built-in MUSCLE (Edgar, 2004) alignment option to construct a consensus tree. The analysis included 71 samples of the control region and mtDNA from our assemblies, as well as 25 different horse breeds deposited in the National Center for Biotechnology Information (NCBI) GenBank database (http://www.ncbi.nlm.nih.gov/). All sequences were processed using blastn v.2.12.0+ (Camacho et al., 2009) to extract an early part of the control region (400 bp in the position between 15,469 and 15,868). The consensus tree (Figure 1D) was built using the Neighbor-Joining method (Saitou and Nei, 1987) with 1,000 bootstrap iterations. The D-loop region sequence of the donkey (*Equus asinus*, NCBI Accession No. NC001788) was used as an outgroup. While phylogeny reconstruction showed Kazakh horse mtDNA sequences are widespread and distributed across many different clusters in the tree, two samples (Kazakh1 and Kazakh5) from the assembled genomes formed a distinct clade with Cheju and Akhal-Teke horses. These results are consistent with previous studies (Gemingguli et al., 2016) reporting tightly linked mtDNA genetic relationships between these breeds. It can be suggested that the Kazakh horse breed has a mixed origin in the maternal lineage, likely due to the use of horse populations in trade and military campaigns, which moved them to distant locations, where they interbred with indigenous populations. The observed lack of Kazakh horse samples clustering in the phylogenetic tree constructed from mtDNA control region sequences may indicate high levels of variability, which, in turn, means that the Kazakh breed may serve as an important reservoir of genetic biodiversity. It is of particular significance for horses, as a species, because its wild ancestors are now extinct and sources of biodiversity that could be used to maintain their functions in certain environments are limited.

## Data Availability

All sequence data presented in this study are deposited in the NCBI Sequence Read Archive (SRA) repository and are publicly available under accession numbers SRX18227458-18227464. The obtained genome assemblies were submitted and registered under the following NCBI GenBank accession numbers: GCA_029814115.1, GCA_029814095.1, GCA_029784105.1, GCA_029814075.1, GCA_029784085.1, GCA_029814055.1.
