# Supplementary material for: Whole genome sequencing and de novo genome assembly of the Kazakh native horse Zhabe
Source: Front Genet. 2024 Oct 21;15:1466382. doi: 10.3389/fgene.2024.1466382 (PMC11551999; doi:10.3389/fgene.2024.1466382)
Supplement: Supplementary file 1 [file Table1.DOCX]

**Supplementary Table S1.** Measurements and live weight of Kazakh horses of the Zhabe type from the LLP agricultural firm «Akzhar Ondiris».

| Sample | Gender | Year of birth | Measurements, cm | | | | Live weight, kg |
| --- | --- | --- | --- | --- | --- | --- | --- |
|  |  |  | Height at withers | Oblique torso length | Chest girth | Pastern girth |  |
| 2H | stallion | 2007 | 148.5 | 157.0 | 189.3 | 22.0 | 530.0 |
| 7H | stallion | 2010 | 147.5 | 156.7 | 193.1 | 20.7 | 518.3 |
| 16H | mare | 2011 | 146.0 | 153.0 | 183.0 | 19.5 | 467.0 |
| 25H | mare | 2014 | 143.0 | 150.0 | 180.0 | 19.5 | 450.0 |
| 30H | stallion | 2010 | 142.0 | 142.0 | 168.0 | 18.5 | 418.0 |
| 57H | mare | 2011 | 144.0 | 151.0 | 181.4 | 19.5 | 459.0 |
