# Supplementary material for: Whole genome sequencing and de novo genome assembly of the Kazakh native horse Zhabe
Source: Front Genet. 2024 Oct 21;15:1466382. doi: 10.3389/fgene.2024.1466382 (PMC11551999; doi:10.3389/fgene.2024.1466382)
Supplement: Supplementary file 2 [file Table2.DOCX]

**Supplementary Table S2.** Summary of sequenced reads.

| Sample | Reads | Bases |
| --- | --- | --- |
| 2H | 5,265,091 | 64,447,046,866 |
| 7H | 3,241,193 | 47,246,743,277 |
| 16H | 10,522,530 | 129,984,183,905 |
| 25H | 3,941,959 | 72,488,404,296 |
| 30H | 11,301,062 | 148,275,765,911 |
| 57H | 5,238,611 | 94,655,288,477 |
