# Supplementary material for: Whole genome sequencing and de novo genome assembly of the Kazakh native horse Zhabe
Source: Front Genet. 2024 Oct 21;15:1466382. doi: 10.3389/fgene.2024.1466382 (PMC11551999; doi:10.3389/fgene.2024.1466382)
Supplement: Supplementary file 3 [file Table3.DOCX]

**Supplementary Table S3.** SNVs and indels identified in assemblies.

| Sample | SNVs | Indels | Ti/Tv |
| --- | --- | --- | --- |
| 2H | 6,970,470 | 613,521 | 1.87 |
| 7H | 6,906,036 | 549,718 | 1.87 |
| 16H | 6,336,129 | 820,662 | 1.95 |
| 25H | 6,796,545 | 621,395 | 1.88 |
| 30H | 7,101,556 | 697,254 | 1.85 |
| 57H | 6,814,522 | 651,441 | 1.87 |
